# Lateralisation of auditory processing in Down syndrome: A study of T-complex peaks Ta and Tb

**DOI:** 10.1016/j.biopsycho.2008.04.003

**Published:** 2008-10

**Authors:** Margriet Anna Groen, Paavo Alku, Dorothy Vera Margaret Bishop

**Affiliations:** a University of Hamburg, Germany; b Helsinki University of Technology, Finland; c University of Oxford, United Kingdom

**Keywords:** Down syndrome, Event-related potential, Auditory, T-complex, Speech sounds, Tones, Lateralisation

## Abstract

It has long been argued that abnormal cerebral lateralisation might underlie the language problems that characterise Down syndrome, but to date only behavioural evidence has been provided. We used the auditory event-related potentials Ta and Tb of the T-complex to investigate lateralised processing of speech (vowels) and non-speech (simple and complex tones) sounds in children with Down syndrome and age-matched typically developing children. We also explored associations with speech and language abilities. Although changes in the Ta and Tb in response to increases in stimulus complexity and ‘speechness’ were similar across group, the Tb peak was delayed in children with Down syndrome across conditions. In addition, marked differences in the patterns of lateralisation of Ta latency and Tb amplitude were observed in children with Down syndrome, in response to both speech and non-speech sounds. No associations were found between Ta and Tb characteristics and speech and language abilities in children with DS.

Language difficulties in people with Down syndrome (DS) are disproportionate in relation to non-verbal ability (Chapman, 1997), and not adequately accounted for by peripheral hearing impairments (Chapman et al., 1991; Marcell and Cohen, 1992; Roizen et al., 1993; Laws, 2004). The language profile in DS is characterised by relatively good performance on tasks assessing vocabulary and pragmatics but relatively poor performance on tasks assessing computational aspects of language such as phonology and grammar (Miller, 1988; Fowler, 1990, 1995; Chapman et al., 1991, 1998; Laws and Bishop, 2003, 2004). Furthermore, receptive language skills are often better than expressive language skills (e.g., Chapman, 1997). As far as speech is concerned, although the presence of anatomical abnormalities in individuals with DS such as a protruding tongue due to a small buccal cavity, hypotonia of speech musculature and chronic upper respiratory tract infections resulting in a lack of nasal resonance are all detrimental to articulation and speech fluency, central deficits in speech movement programming are necessary to explain important characteristics of speech in this group, including their inconsistency in speech errors (Frith and Frith, 1974; Dodd, 1976; Dodd and Thompson, 2001). This profile has led to direct comparisons with children with Specific Language Impairment (SLI), who show deficiencies in language development that cannot be explained by low non-verbal cognitive ability, abnormalities in sensory abilities or other neurological or psychiatric conditions (Bol and Kuiken, 1990; Eadie et al., 2002; Laws and Bishop, 2003).

In other clinical conditions, such as SLI and dyslexia, it has been suggested that an impairment in basic auditory processing might contribute to the language and literacy difficulties (e.g., Tallal and Piercy, 1973; Tallal, 1976; Wright et al., 1997; McArthur and Bishop, 2004; Hill et al., 2005). One question is whether deficits in auditory processing may also contribute to the language difficulties observed in children with DS. Although very little is known about auditory processing skills in individuals with DS, there is some evidence for impairments in speech perception in this group. For instance, young people with DS had higher thresholds for speech, took longer to process it and needed more information before correctly identifying it than a group of individuals with intellectual disabilities due to other aetiologies (Marcell and Cohen, 1992). Furthermore, results from dichotic listening studies suggest a lack of typical left hemisphere specialisation for speech perception in individuals with DS. In a dichotic listening paradigm, two different auditory stimuli (e.g., words or syllables) are presented at the same time, one in each ear. The participant is asked to report the stimulus heard first or best. In healthy controls, a right-ear advantage for speech stimuli in this procedure is taken as a behavioural measure of left hemisphere specialisation for speech perception. In the original model proposed by Kimura (1967) it is suggested that this right-ear advantage for speech is caused by several interacting factors. Information to each ear is better represented in the opposite hemisphere via the dominant contralateral auditory pathways, and right-ear linguistic input, therefore, has a stronger connection to the language centres in the left hemisphere. Moreover, information carried in the ipsilateral auditory pathways is inhibited by unrelated activation of the contralateral pathways. Finally, speech input to the left-ear is subject to delay as it crosses from the right to the left hemisphere via the corpus callosum. In individuals with DS a left-ear right-hemisphere preference for speech sounds (Hartley, 1981; Pipe, 1983; Bowler et al., 1985; Elliott and Weeks, 1993) or no ear advantage (Sommer and Starkey, 1977; Tannock et al., 1984) has been observed, instead of the typical right-ear left-hemisphere preference found in healthy controls.

Assessing auditory processing abilities in individuals with DS is complicated by their limited cognitive abilities. An alternative method to investigate basic auditory processing and the lateralisation of these processes, that is not influenced by variations in listening strategy or concentration, is provided by examining the brain's responses to sounds. The series of auditory event-related potentials known as the T-complex is particularly useful as it is clearly lateralised in typically developing individuals. First described by Wolpaw and Penry (1975), the T-complex consists of a series of peaks in the 80–160 ms latency range in adults, recorded at temporal scalp locations. A positivity at about 100 ms, Ta, is followed by a negativity at approximately 150 ms, Tb (also referred to as N1c; McCallum and Curry, 1980). The neural generators of Ta are not well defined at present, but dipole models have attributed Tb activity to bilateral radial dipoles in the temporal lobe (Scherg and Von Cramon, 1985, 1986; Ponton et al., 2002). In addition, evidence from intra-cranial recordings and lesion studies suggest that Tb reflects activity from the lateral surfaces of the temporal lobe, that is, secondary auditory cortex (Celesia et al., 1968; Celesia and Puletti, 1969; Peronnet et al., 1974). Both Ta and Tb are present from an early age and decrease in latency and amplitude with development up to 12–16 years of age (Gomes et al., 2001; Tonnquist-Uhlen et al., 2003). Indeed, in children these temporal peaks are more prominent than fronto-central components (Gomes et al., 2001; Bruneau et al., 1997). In contrast to the fronto-central peaks, the T-complex peaks appear less sensitive to stimulus rate as they can be recorded in young children even with fast stimulus presentation (Gomes et al., 2001). Furthermore, with monaural stimulation, the Ta and Tb occur earlier and have bigger peaks on the side contralateral to the ear of presentation in adults (Wolpaw and Penry, 1975; Connolly, 1985, 1993; Cacace et al., 1988) and children (Tonnquist-Uhlen, 1996; Tonnquist-Uhlen et al., 2003). As far as we are aware, only one study has explored modulation of the Ta and Tb peaks as a function of ‘speechness’, although it should be noted that the tone and the vowel were not matched in complexity in this study. Eulitz et al. (1995) reported that the latencies of the Ta and the Tb in adults were prolonged and the Tb amplitude smaller in response to a vowel, compared to a simple tone. Finally, in other clinical populations with language impairments such as children with SLI and children with autism, a high incidence of delayed, deviant or absent T-complex peaks has been reported (Bruneau et al., 1999; Tonnquist-Uhlen, 1996; Mason and Mellor, 1984). As far as we are aware, the Ta and Tb have not been described in individuals with DS.

Here we report data on the Ta and Tb elicited by speech and non-speech sounds in children with DS, and explore their associations with speech and language abilities in these children. Specifically, we set out to shed light on the following questions. First, do children with DS show a higher incidence of delayed, deviant or absent Ta and Tb peaks, as has been reported in children with SLI and children with autism? Second, are there differences in Ta and Tb in response to speech sounds compared to tones? Third, do children with DS show atypical patterns of lateralisation of auditory processing as reflected by abnormal lateralisation patterns of Ta and/or Tb? We presented speech and non-speech sounds matched in stimulus complexity and hypothesised that an atypical pattern of lateralisation in children with DS might be more pronounced in response to speech sounds compared to non-speech sounds. Fourth, are abnormalities in the appearance or lateralisation of Ta and/or Tb associated with language deficits in children with DS? As several studies have found that hand preference is less strongly developed in individuals with DS (Cornish et al., 1997; Carlier et al., 2006; Groen et al., 2008) and weaker hand preference (i.e., less consistent use of the left or right hand) has been associated with weaker language skills in children with DS (Groen et al., 2008), we also included measures of hand preference and examined associations between the consistency in hand preference and lateralisation of the Ta and Tb.

## 1 Methods and materials

### 1.1 Participants

Nineteen typically developing children (13 girls) and 19 children with DS (11 girls) aged 10–12 years were recruited for this study. The children with DS were recruited through local and national support groups. The typically developing children came from local primary schools and were age-matched to the DS group. English was the main language spoken at home for all children and informed parental consent was obtained for all of them. Two children with DS refused to wear the electrode cap, and one child with DS and one typically developing child had large amounts of movement or muscle artefact in the EEG record. Moreover, the T-complex could not be identified on both T7 and T8 in 3 children with DS and 2 typically developing children. Finally, children with an average hearing threshold >25 db HL across speech frequencies (0.5, 1, 2, and 4 kHz) were excluded from the study. In the typically developing children, a screening procedure was used to pick up children with hearing thresholds >25 dB HL on speech frequencies. None of the typically developing children exceeded this criterion on any of the frequencies. For the children with DS, exact hearing thresholds were established. Three children with DS had an average hearing threshold across speech frequencies >25 dB HL and were excluded from the study. This resulted in 16 typically developing children and 10 children with DS with complete data sets. These 10 children with DS had an average hearing threshold across speech frequencies of 14.8 dB (range: 1.3–22.5). Maximum hearing thresholds on any individual frequency did not exceed 30 dB in these children with DS.

### 1.2 Behavioural measures

#### 1.2.1 Leiter-R

Two subtests (Sequential Order and Repeated Patterns) of the non-verbal IQ test, Leiter International Performance Scale-Revised (Roid and Miller, 1997) were used to derive a ‘Fluid Reasoning IQ’ score, which has good reliability (Cronbach's alpha = .89).

#### 1.2.2 Hand preference

A card-reaching task (Bishop et al., 1996) provided a behavioural measure of the strength, in addition to the direction, of hand preference. In the card-reaching task, stacks of three picture cards were placed in seven spatial locations (approximately 30° apart) along a semi-circle within the child's reach. The child was seated in the centre of the semi-circle and asked to pick up a specific card and place it in a box located directly in front of them, without time constraints. The same random sequence of positions was used for all participants. The child was not informed of the experimental interest in hand preference. The following dependent variables were computed from performance on the card-reaching task. Firstly, a laterality index (LI) was computed by subtracting 0.50 from the proportion of right-hand reaches. This score ranged from +0.50 for participants reaching exclusively with the right hand through 0 for children who did not show a preference to −0.50 for those reaching exclusively with the left. Secondly, as we were particularly interested in differences between children with and without a clear hand preference, the ‘strength’ of hand preference was determined by taking the absolute value of the laterality index. In this way, children with a strong preference for the left or right hand received a maximum score of .50.

#### 1.2.3 CCC-2

To obtain an estimate of the child's use of language in everyday situations and assess aspects of communication that are not easily evaluated using more traditional language tests, parents completed the Children's Communication Checklist - 2nd edition (CCC-2; Bishop, 2003). This questionnaire consisted of 70 multiple-choice items, divided into 10 scales. The first four scales (speech, syntax, semantics and coherence) assessed aspects of language structure, vocabulary and discourse. The next four scales (inappropriate initiation, stereotyped language, use of context and non-verbal communication) covered pragmatic aspects of communication. The last two scales (social relations and interests) assessed behaviours that are usually impaired in cases of autistic disorder. The score reported here is the general communication composite score (GCC), which consists of the sum of the scaled scores for the first eight scales. A GCC score below 55 is representative of the bottom 10% of children in the normative sample and virtually all children with a diagnosis of SLI or autism obtain a score lower than that.

#### 1.2.4 Articulation and oro-motor skills

The Diagnostic Screen subtest of the Diagnostic Evaluation of Articulation and Phonology (DEAP; Dodd et al., 2002) was used to assess accuracy of articulation. We report here the average percentage of consonants produced correctly over two trials of naming 10 pictures. Oro-motor skills were assessed using the Speech rate subtest of the Phonological Abilities Test (PAT, Muter et al., 1997). This subtest required the child to repeat the word ‘buttercup’ ten times, as quickly as possible while the examiner recorded the time taken.

#### 1.2.5 Vocabulary

Receptive vocabulary was assessed using the British Picture Vocabulary Scale - 2nd Edition (BPVS-2; Dunn et al., 1997). The child was asked to identify, from four choices, the illustration that best depicted the meaning of a word presented orally by the experimenter.

### 1.3 Auditory ERPs

#### 1.3.1 Stimuli

Stimuli were created using the semi-synthetic speech generation method, (SSG; Alku et al., 1999) which creates natural sounding speech stimuli by exciting an artificial vocal tract model with a real excitation of the human voice production mechanism, the glottal waveform. With this methodology, the glottal excitation was first computed from a sustained vowel/e/produced by a native male speaker of British English. Then, the same speaker was asked to produce a series of the word “bard” and the vocal tract was estimated with SSG during the vowel/a/in one of the words repeated. Recordings were conducted in an anechoic chamber with a high-quality condenser microphone (AKG C444) and sounds were saved directly onto a digital hard-disk player (iRiver H140). The vowel /a/was generated by filtering the natural glottal excitation through the estimated vocal tract model. The most important acoustical cues of the vowel, the lowest four formants, were then extracted from the vocal tract model obtained. Using this information, both the simple tone and complex tone stimulus were created by generating sinusoidals, the frequencies and intensity levels of which matched the strongest harmonics of the vowel/a/in the vicinity of the extracted four formants. This procedure yielded the frequency of 576 Hz for the simple tone. The complex tone, in turn, was composed of four sinusoidals located at 576, 1055, 2589 and 3163 Hz. Finally, the lengths of the stimuli were set to 300 ms (including 25 ms rise time, 200 ms plateau and 75 ms fall time). Stimuli were presented with equal probability and in a randomised order with an inter-stimulus interval of 1550 ms on average, jittered with a S.D. of 30 ms. Absolute sound pressure levels at which the sounds were presented were scaled individually to correspond to 50 dB SPL (a-weighted), by using a Brüel and Kjær sound level meter type 2260 with a Brüel and Kjær artificial ear 4152 and a polarised microphone type 4144. They were presented monaurally to the right-ear, using ER-3A insert earphones.

#### 1.3.2 Recordings

The EEG was recorded from 21 sintered Ag/AgCl electrodes placed according to the 10–10 system (American Electroencehalographic Society, 1994; Fp1, Fp2, F7, F3, Fz, F4, F8, FCz, T7, C3, Cz, C4, T8, P7, P3, Pz, P4, P8, O1, O2, and the right mastoid), using a Neuroscan Nuamps amplifier and SCAN 4.3 acquisition software. The left mastoid was used as the online reference, and AFz as the ground electrode. Eye-blinks were monitored by recording a vertical electro-oculogram (VEOG), using bipolar electrodes above and below the left eye in all typically developing children and in eight children with DS. As has been reported by others (Schafer and Peeke, 1982), some individuals with DS find electrodes in the face particularly disturbing, and the remaining two children with DS in this study did not tolerate the recording of a VEOG. The signal was sampled at 1000 Hz and band-pass filtered between 0.1 and 70 Hz. Impedances were kept below 10 kΩ. Participants were comfortably seated in the sound attenuated part of a research van, and watched a subtitled DVD on a 17.5 cm screen placed approximately 30 cm in front of them. The sound was turned off for all, except four children with DS for whom the sound was turned on very softly (barely audible) to keep them happy. To rule out the possible confound of an audible soundtrack in one group in the current study, analyses were repeated using only the data from children with DS for whom the DVD soundtrack was off (*n* = 6). As this did not change the results, we will report results including all children with DS (*n* = 10) to increase power.

#### 1.3.3 Analysis

Analyses were carried out using Neuroscan's SCAN 4.3 analysis software. Because the VEOG was not available for all children with DS and to be consistent across participant groups, the continuously recorded EEG on Fp1 was used to deal with eye-blink artifacts, using the ‘Ocular artifact reduction’ procedure offered in SCAN 4.3. An electrode above the eye or Fp1, instead of a bipolar montage, has been used by several groups to monitor eye-blinks, and was found to yield a similar eye-blink waveform (Callner et al., 1978; Kraus and McGee, 1993; Wunderlich et al., 2006). Our own analyses with data from typically developing children for whom both were available confirmed that no systematic differences across conditions were introduced by using Fp1 instead of the VEOG to correct for eye-blinks.

Recordings were divided into 700 ms epochs, including a 200 ms pre-stimulus interval over which epochs were baseline corrected. Trials containing EEG activity greater than ±125 μV on any electrode were rejected. Average numbers of trials included for the simple tone, complex tone and vowel condition were 93, 91, and 93 for the typically developing children and 88, 90, and 90 for the children with DS, respectively. Stimulus-locked averages of trials of the same condition were created, and re-referenced to an average reference of all electrodes after a zero-phase shift low-pass filter was applied using a half-amplitude cut-off frequency of 30 Hz and a net roll-off of 96 dB/octave.

Following Luck (2005), local peak amplitude and latency were determined for the Ta and Tb peak at T7 and T8 from the averaged waveforms for each individual. For Ta the first positive peak between 70 and 170 ms was taken. For Tb, the following negative peak, falling in the time window of 150–250 ms, was taken. Peak amplitude was measured relative to the averaged amplitude over the pre-stimulus interval. Separate repeated measures ANOVAs for the latency and the amplitude measures for each peak were carried out with Stimulus (3 levels: simple tone, complex tone, vowel) and Hemisphere (2 levels: T7, T8) as the within-subjects factors and Group (2 levels: typically developing children, children with DS) as the between-subjects factor. Where Mauchly's test indicated that the assumption of sphericity was violated, the Greenhouse–Geisser correction was used. In that case the results presented indicate successively the uncorrected degrees of freedom, the *F* value, the epsilon-corrected *p* value and the corresponding epsilon value. Significant main effects with more than two levels were further examined using planned contrasts of the ‘repeated’ type, in which each category, except the first, is compared to the previous category (simple tone vs. complex tone and complex tone vs. vowel). Significant interactions were followed up by appropriate *t*-tests. In addition, Pearson's correlation coefficient *r* is reported as a measure of effect size when comparing two means (Field, 2005). Following Cohen (1992), *r* = .10 is considered a small, *r* = .30 a medium, and *r* = .50 a large effect.

To assess associations between T-complex peak characteristics and behavioural measures, two approaches were taken. First, Pearson correlation coefficients were computed between behavioural measures and characteristics of the Ta and Tb. To control the Type I error rate for these correlations, the significance level was set at *p* ≤ 0.01. Second, language profiles of individual children with DS were considered.

## 2 Results

### 2.1 Behavioural measures

Performance of typically developing children and children with DS on the card-reaching task, and the speech and language assessments is summarised in Table 1. As would be expected, in comparison with age-matched typically developing controls, the DS group obtained scores that were lower by several S.D. units on speech and language assessments. On the card-reaching task, typically developing children demonstrated a significantly more positive LI than the children with DS (*U*^1^ = 33.00, *p* = .011, *r* = −.50), indicating that typically developing children used their right hand more often than the children with DS. In addition, independent of the hand (left or right) used, typically developing children showed a stronger hand preference on the card-reaching task. That is, they used the same hand more often to pick up cards than children with DS (*U* = 38.50, *p* = .022, *r* = −.45).

The typically developing children showed non-verbal reasoning, speech and language skills in the normal range, whereas the children with DS scored well below the normal range on all tests.

### 2.2 Auditory event-related potentials

Absence of T-complex peaks was observed in similar percentages of children with DS and typically developing children (Fisher's Exact test = .625). In three (out of 13 = 23%) children with DS and two (out of 18 = 11%) typically developing children with ERP data without large amounts of artifacts, the Ta and Tb peaks could not be identified on both T7 and T8.

**Fig. 1:**
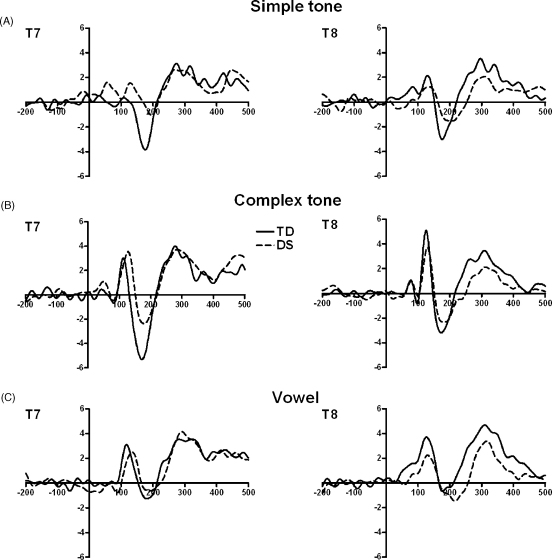
Event-related potential waveforms on T7 and T8 for typically developing children (TD) and children with Down syndrome (DS) in response to (A) simple tones, (B) complex tones, and (C) vowels. Amplitude (μV) is plotted on the *Y*-axis, time (ms) on the *X*-axis. Negative is plotted downwards. Stimulus onset was at time 0.

Grand average waveforms in response to simple tones, complex tones and vowels for typically developing children and children with DS with measurable Ta and Tb peaks on both T7 and T8 are depicted in Fig. 1.

#### 2.2.1 Ta

**Fig. 2:**
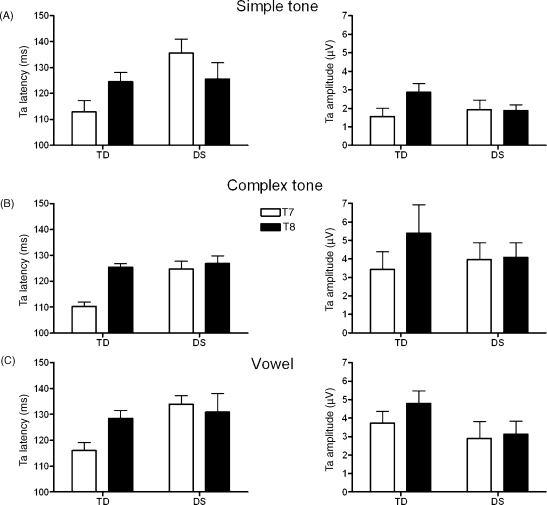
Mean latencies and amplitudes for the Ta on T7 (open bars) and T8 (solid bars) in typically developing children (TD) and children with Down syndrome (DS) in response to (A) simple tones, (B) complex tones, and (C) vowels. Errors bars represent standard errors of the mean.

Mean latencies and amplitudes for Ta are shown in Fig. 2. The latency of the Ta peak was not influenced by stimulus condition as indicated by the absence of a significant main effect of Stimulus or any interactions with the Stimulus factor. However, a significant main effect of Group (*F*(1,24) = 7.88, *p* = .010, *r* = .50) was modified by a significant Hemisphere by Group interaction (*F*(1,24) = 10.50, *p* = .003), indicating a significantly shorter latency in typically developing children compared to children with DS on T7 (*t*(24) = −4.19, *p* = .000, *r* = .65), but not on T8 (*t*(24) = −0.37, *p* = .715, *r* = .01). In other words, a shorter Ta latency on the contralateral electrode T7 compared to the ipsilateral electrode T8 was found in typically developing children (*t*(15) = −6.20, *p* = .000, *r* = .85), but not in children with DS (*t*(9) = 0.65, *p* = .535, *r* = .21).

For Ta amplitude a significant main effect of Stimulus was found (*F*(2,48) = 8.99, *p* = .002, *ɛ* = .73). Ta amplitude was smaller in response to simple tones than in response to complex tones (*F*(1,24) = 10.92, *p* = .003, *r* = .56) in both groups, but did not differ when comparing complex tone- and vowel-elicited responses (*F*(1,24) = 1.21, *p* = .282, *r* = .22) in either group. No effects of Hemisphere or Group were found for the Ta amplitude.

#### 2.2.2 Tb

**Fig. 3:**
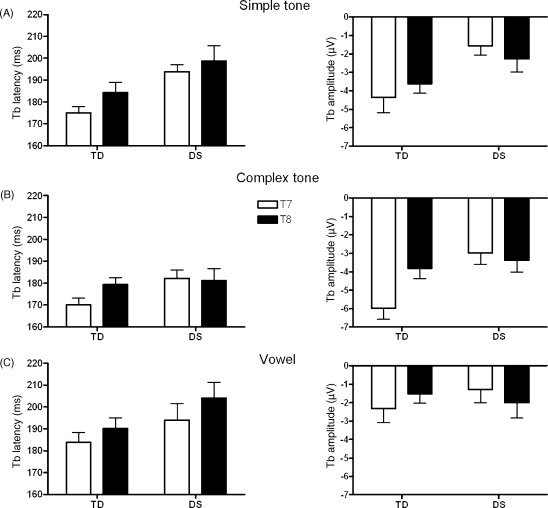
Mean latencies and amplitudes for the Tb on T7 (open bars) and T8 (solid bars) in typically developing children (TD) and children with Down syndrome (DS) in response to (A) simple tones, (B) complex tones, and (C) vowels. Errors bars represent standard errors of the mean.

Mean latencies and amplitudes for Tb are shown in Fig. 3. Significant main effects of Stimulus were found for Tb latency (*F*(2,48) = 10.87, *p* = .000) and amplitude (*F*(2,48) = 16.49, *p* = .000). Repeated contrasts showed that Tb occurred earlier in response to complex tones than in response to simple tones (*F*(1,24) = 9.85, *p* = .004, *r* = .54) or vowels (*F*(1,24) = 19.29, *p* = .000, *r* = .67) in both groups. Tb was also bigger in response to complex tones compared to simple tones (*F*(1,24) = 9.29, *p* = .006, *r* = .53) and vowels (*F*(1,24) = 44.75, *p* = .000, *r* = .81) in both groups.

In addition, the Tb peak was delayed in children with DS by about 12 ms compared to their typically developing peers as indicated by a significant main effect of Group (*F*(1,24) = 13.97, *p* = .001, *r* = .61). Also a trend towards a significant main effect of Hemisphere suggested a shorter latency for the Tb peak on T7 compared to T8 in both groups (*F*(1,24) = 3.61, *p* = .070, *r* = .36).

A significant main effect of Group for Tb amplitude (*F*(1,24) = 5.05, *p* = .034, *r* = .42) was modified by a significant Hemisphere by Group interaction which fell just short of significance (*F*(1,24) = 3.78, *p* = .064). This trend was explored further using *t*-tests, which indicated that Tb was bigger in typically developing children than in children with DS on T7 (*t*(24) = −2.79, *p* = .010, *r* = .49), but not on T8 (*t*(24) = −0.61, *p* = .547, *r* = .12). Another way of describing this result is that there was a trend for a bigger Tb contralaterally than ipsilaterally in typically developing children (*t*(15) = −1.93, *p* = .072, *r* = .45), but not in children with DS (*t*(9) = 0.98, *p* = .354, *r* = .31).

### 2.3 Associations between T-complex peak characteristics and behavioural measures

For several characteristics of T-complex peaks, group differences were observed. These characteristics were used to explore correlations with behavioural measures. First, the average delay of the Tb peak in children with DS relative to the typically developing children across conditions was computed and Pearson correlation coefficients of this delay with behavioural measures were examined. No significant correlations were found. Of interest is that the delay in Tb latency also did not correlate significantly with hearing level (*r*(10) = .05, *p* = .89), indicating that hearing levels alone are insufficient to explain the prolonged Tb latencies in children with DS.

**Fig. 4:**
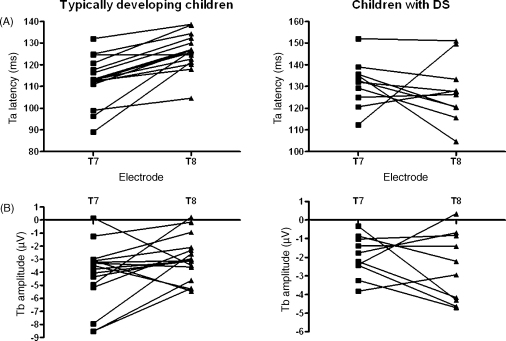
Individual participants’ values for (A) Ta latency, and (B) Tb amplitude on T7 and T8 for typically developing children (left) and children with Down syndrome (right). Whereas the typically developing children showed shorter Ta latencies and bigger Tb amplitudes on T7 compared to T8, this was not the case for most children with Down syndrome.

Second, two differences in the lateralisation of the Ta and Tb were observed in children with DS compared to typically developing controls. In typically developing children, the latency of the Ta peak was on average 13 ms shorter on T7 compared to T8, whereas in children with DS no significant difference was found (Fig. 4A). Tb amplitude was on average 1.2 μV bigger on T7 compared to T8 in typically developing children, but not in children with DS (Fig. 4B). We used these difference scores to explore any associations between the degree of lateralisation of T-complex peaks and behavioural measures in two ways. Pearson correlation coefficients were examined in typically developing children and children with DS separately. No significant correlations were found in either group. Further consideration of individual children who either had absent T-complex peaks, or conversely, who looked normal on these peaks in terms of lateralisation, did not indicate any pattern of association with behavioural measures (either with language, non-verbal cognitive ability and memory or handedness measures).

## 3 Discussion

### 3.1 Characteristics of the Ta and Tb in response to simple tones, complex tones and vowels

Unlike findings reported for children with SLI and autism, Ta and Tb were present in a similar percentage of children with DS and typically developing children. However, Tb was delayed in children with DS across stimulus conditions. This was the case even though all children had hearing levels in the normal range. It is unlikely that the increase in Tb latency in children with DS is the consequence of a difference in attention to the auditory stimuli. The single study that examined the modulation of T-complex peaks by attention found an overall increase in Tb amplitude when tones were attended, but no differences in Tb latency (Hackley et al., 1990). Alternatively, the prolonged latencies of auditory potentials in individuals with DS might be associated with deficits in myelination or as a result of thyroid dysfunction. With regard to the first, findings from several groups suggest that deficits in myelination might be present in individuals with DS (e.g., Banik et al., 1975; Wisniewski and Schmidt-Sidor, 1989; Krapfenbauer et al., 2001; Pinter et al., 2001; Vlkolinsky et al., 2001). With regard to the latter, thyroid dysfunction is common in individuals with DS, taking the form of hypothyroidism at both clinical and subclinical levels (Dinani and Carpenter, 1990; Ivarsson et al., 1997; Prasher and Gomez, 2007) and other auditory ERPs (P1 and N1b) were found to be delayed in a group of young adults with severe congenital hypothyroidism who did not have Down syndrome (Oerbeck et al., 2007). These potential explanations for prolonged latencies of auditory ERPs in individuals with DS should be examined in future work.

The changes in the T-complex peaks as a function of stimulus were similar across groups. In typically developing children and children with DS, Ta and Tb amplitude were bigger and Tb latency was shorter in response to complex tones compared with responses to simple tones. Wider activation in response to complex compared to simple tones has been reported in both animals and humans (Rauschecker, 1998; Schreiner, 1998; Wessinger et al., 2001; Hall et al., 2002). Similarly, the bigger amplitudes of Ta and Tb and the shorter latency of Tb in response to the complex tone in the current study might be a reflection of either the activation of additional cell networks responsive to higher frequencies or the synchronous activation of additional cell networks involved in more advanced stimulus processing, or a combination of the two. In this respect it would be of interest to use stimuli with similar spectral complexity, but differing in other aspects of acoustical complexity, such as its temporal structure, in a future study. If the shorter latency and bigger amplitudes ‘merely’ reflect the wider range of frequency channels activated by the complex tone, one would not see a similar change in response to a temporally more complex tone. Both groups also showed a longer Tb latency and smaller Tb amplitude in response to vowels compared to complex tones. This finding is in agreement with the results reported by Eulitz et al. (1995) in adults. Because the stimuli in that study were not matched in complexity, the present result adds that the speech sound quality rather than the stimulus complexity is associated with these changes in the Tb. Together, these findings suggest that the neural generators underlying the Ta and Tb are similarly sensitive to changes in stimulus complexity and ‘speechness’ in both groups.

### 3.2 Lateralisation of Ta and Tb

Marked differences between children with DS and typically developing children were found in terms of the laterality patterns of the T-complex peaks. In accordance with the literature (Wolpaw and Penry, 1975; Connolly, 1985, 1993; Cacace et al., 1988; Tonnquist-Uhlen, 1996; Tonnquist-Uhlen et al., 2003) a shorter latency for the Ta peak and a trend for a bigger amplitude for the Tb peak at the side contralateral to the ear of stimulation compared to the ipsilateral side were found in typically developing children. In contrast, no significant differences between Ta latency and Tb amplitude on contra- vs. ipsilateral sides were observed in children with DS. This was the case across stimulus conditions; therefore, we did not find evidence to support our hypothesis that abnormal lateralisation would be more pronounced in response to speech sounds. Nevertheless, this finding supports the idea that lateralisation of auditory processing is atypical in children with DS.

Little is known about the origins of the lateralisation of the Ta and Tb peaks observed in typically developing individuals. However, it fits well with the general finding that contralateral projections are larger, more preponderant and relay information faster than the ipsilateral projections in the auditory system (e.g., Tunturi, 1946; Ades and Brookhart, 1950; Rosenzweig, 1951), and there is some evidence to suggest that transfer of information via the corpus callosum contributes to the asymmetry. Specifically, Aiello et al. (1994) recorded T-complex peaks in response to clicks and tones presented monaurally and binaurally to a patient with complete corpus callosum agenesia. The Ta and Tb peaks were present over both hemispheres when stimuli were presented binaurally. However, in the case of monaural stimulation, peaks at the side contralateral to the ear of stimulation were observed to be normal, whereas peaks at the side ipsilateral to the ear of stimulation were described as ‘of abnormal shape’, ‘smaller in amplitude’ and ‘in some instances completely abolished’ (Aiello et al., 1994, p. 503). The authors conclude that the observed weak ipsilateral potentials in response to monaural stimulation originate from ipsilateral pathways that are inhibited by the contralateral pathways, and infer that well-formed Ta and Tb peaks over both hemispheres in response to monaural stimulation can occur only if transfer of auditory inputs via the commissural fibres is possible. Further support for the idea that transfer via the corpus callosum plays a role in the asymmetry observed in the T-complex peaks in response to monaural stimulation comes from work by Cracco et al. (1989). They showed that human transcallosal responses elicited by means of a focal magnetic coil had a latency of 9–12 ms. This would correspond well to the 13 ms difference in Ta latency between T7 and T8 observed in the current study and the 8–20 ms difference found in other studies (e.g., Cacace et al., 1988; Connolly, 1993; Tonnquist-Uhlen et al., 2003).

Although abnormalities in the corpus callosum have been reported in individuals with DS (e.g., Wang et al., 1992), two aspects of the results suggest that this abnormal lateralisation is due to deficits that affect mainly the contralateral auditory pathways and might, therefore, not involve the corpus callosum. Firstly, the absence of a difference in Ta latency at T7 vs. T8 in children with DS resulted from prolonged latencies at the contralateral electrode T7, whereas the latencies at the ipsilateral electrode T8 were similar to those observed in typically developing children. Similarly, the lack of a difference in Tb amplitude at T7 vs. T8 resulted from a smaller Tb amplitude at T7, whereas Tb amplitude at T8 was similar to the one observed in their typically developing peers. Following the results reported by Aiello et al. (1994), if the corpus callosum would be involved in the atypical patterns of lateralisation of the Ta and Tb in children with DS one would have expected the ipsilateral, rather than the contralateral T-complex peaks to show abnormalities.

A different mechanism that might be involved in the abnormal lateralisation of the Ta latency observed in children with DS is abnormalities in neurotransmitter systems. Wolpaw and Penry (1978) reported that the difference in Ta latency at contra- vs. ipsilateral temporal electrodes could be modulated by the consumption of ethanol and caffeine in healthy adults. Both ethanol and caffeine consumption resulted in increases in the Ta latency difference. However, ethanol consumption increased the latency difference by selectively increasing the ipsilateral Ta latency, whereas caffeine consumption increased the latency difference by selectively decreasing the contralateral Ta latency. As caffeine consumption increases the amount of extracellular dopamine in the brain and has also been reported to affect acetylcholine and serotonin metabolism (e.g., Nehlig et al., 1992), one could infer that the lack of Ta latency lateralisation in children with DS reflects abnormalities in these neurotransmitter systems. Indeed, several studies have reported deficits in neurotransmitter systems in individuals with DS (e.g., Fodale et al., 2006; Seidl et al., 1999). In future work it would be of great importance to explore the associations between functioning of these three neurotransmitter systems and Ta latency asymmetry further in both typical individuals and individuals with DS.

Finally, two alternative explanations for differences in Ta and Tb lateralisation in children with DS and typically developing children need to be considered. First, could subtle differences in hearing thresholds between the children with DS and the typically developing children explain the observed differences in Ta and Tb lateralisation? This seems unlikely as Cacace et al. (1988) reported that contra- vs. ipsilateral differences in T-complex peaks were not influenced by stimulus intensity. Even at soft levels (e.g., 20 dB SL), the differences between contra- and ipsilateral sites remained. Secondly, could differences in attention to the auditory stimuli explain the observed differences in Ta and Tb lateralisation? Kinsbourne (1978) proposed that asymmetries observed in dichotic listening paradigms could be explained by a cognitive or attentional bias toward the hemispace contralateral to the engaged hemisphere. As Hackley et al. (1990) used the right mastoid as a reference electrode in their study on the effects of attention on T-complex peaks, differences between T-complex peaks at contra- and ipsilateral electrodes could not be evaluated. It is, therefore, an empirical question whether the difference in Ta latency or Tb amplitude at contra- vs. ipsilateral sites is affected by attention and whether the differences are dependent on the affinity of the hemisphere with the auditory material presented.

### 3.3 Associations between Ta and Tb characteristics and language ability in children with DS

Overall, we did not find evidence for our hypothesis that abnormalities in central auditory processing as reflected in the Ta and Tb might be associated with severity of language deficits in children with DS. No significant correlations were found between behavioural measures and either the delay of the Tb or the degree of lateralisation of the Ta or Tb in the current sample. Equally, the comparisons of language profiles of individual children with DS who did not show measurable Ta and Tb peaks on both temporal electrodes, or children with DS who showed a pattern of Ta and Tb lateralisation similar to typically developing children, did not point to associations between Ta and Tb characteristics and language abilities. It should be noted though that the sample is rather small for a comprehensive evaluation of individual differences, and all children with DS had severe language impairments. The restricted range of language skills in the DS group could limit the power to detect correlations. Also we should note that two of the children with DS without measurable T-complex peaks were among the youngest in the group. Although the T-complex peaks have been reported in much younger typically developing children (from age 5 years onwards, e.g., Tonnquist-Uhlen et al., 2003), it would be of interest to include younger children with DS in a future study.

### 3.4 General conclusions and directions for future work

In this study, we found many similarities between auditory ERPs of children with DS and age-matched controls, despite substantial differences in cognitive ability. Ta and Tb were present in a similar proportion of children in both groups, and changes in the Ta and Tb in response to increases in stimulus complexity and ‘speechness’ were very similar across groups. However, there were two striking features seen in the children with DS: the Tb peak was delayed, and there were marked differences in the patterns of lateralisation of Ta latency and Tb amplitude. These features were seen in response to both speech and non-speech sounds, indicating a general rather than a speech-specific abnormality in auditory processing in children with DS. A limitation of the current study was that stimuli were presented monaurally to the right-ear only. Contra- and ipsilateral responses were, therefore, confounded with possible effects of hemisphere. In future work, it would be important to compare T-complex peaks in response to right- and left-ear monaural stimulation, and possibly also binaural and dichotic stimulation. In that way, contra- and ipsilateral pathways in both hemispheres could be investigated and effects of hemisphere could be examined separately. A second limitation of this study is that we studied only one clinical group, so it is unclear whether the delay in Tb latency and/or the abnormal pattern of lateralisation of Ta and Tb is characteristic of individuals with DS, or whether it is a result of low general cognitive ability. Comparing responses of individuals with DS with those of another group of individuals of similar general cognitive ability, but with a different aetiology would be informative: it might be especially interesting to contrast Down syndrome with Williams syndrome, given the contrasting neuroanatomical profiles of these two disorders (Wang et al., 1992). Furthermore, to better understand the abnormalities in the Ta and Tb in individuals with DS, more comprehensive knowledge of characteristics, dependencies and underlying sources of Ta and Tb in typical individuals is needed. In particular, the way the Ta and Tb are modulated by different neurotransmitter systems, and the roles of the corpus callosum and attention in the observed laterality patterns of the T-complex peaks need clarifying. Finally, of great interest would also be the degree to which abnormal Ta and Tb lateralisation is associated with atypical ear preferences in dichotic listening paradigms.

In sum, this is the first study of lateralisation for processing of sounds in DS using an electrophysiological method. The results show convergence with previous behavioural reports in demonstrating lack of cerebral lateralisation in this group.

## Figures and Tables

**Table 1:** Performance on behavioural measures for typically developing children (TD) and children with Down syndrome (DS) with measurable T-complex peaks

	TD (*n* = 16)	DS (*n* = 10)
Age (months)	133.75 (7.05)	135.50 (5.06)
Leiter fluid reasoning IQ^a^	94.31 (11.79)	58.50 (5.93)
Card-reaching task LI	0.48	0.07
Card-reaching task strength	0.50	0.17
CCC-2 GCC^b^	63.07 (16.00)	23.44 (12.13)
BPVS-2^a^	102.25 (12.23)	56.60 (13.04)
DEAP screen (% consonants correct)	99.50 (1.37)	74.40 (15.23)
PAT speech rate (words/s)	1.42 (0.29)	0.83 (0.25)

Means (standard deviations) are reported for all measures except those derived from the card-reaching task, in which case the Median is reported. *Note*: LI = Laterality Index. CCC-2 = Children's Communication Checklist (2nd Edition). GCC = General Communication Composite. BPVS-2 = British Picture Vocabulary Test (2nd Edition). DEAP Screen = Diagnostic Screen of the Diagnostic Evaluation of Articulation and Phonology. PAT = Phonological Abilities Test.

a Standard score *M* = 100, S.D. = 15.

b TD *n* = 14, DS *n* = 9.

## Footnotes

1 Non-parametric statistics were used for the comparisons of performance on the card-reaching task as the data were heavily skewed, in particular in the typically developing children.

## References

[bib1] Ades H.W., Brookhart J.M. (1950). The central auditory pathway. Journal of Neurophysiology.

[bib2] Aiello I., Sotgui S., Sau G.F., Manca S., Conti M., Rosati G. (1994). Long latency evoked potentials in a case of corpus callosum agenesia. Italian Journal of Neurological Science.

[bib3] Alku P., Tiitinen H., Naatanen R. (1999). A method for generating natural-sounding speech stimuli for cognitive brain research. Clinical Neurophysiology.

[bib4] American Electroencehalographic Society (1994). Guideline thirteen: guidelines for standard electrode position nomenclature. Journal of Clinical Neurophysiology.

[bib5] Banik N.L., Davison A.N., Palo J., Savolainen H. (1975). Biochemical studies on myelin isolated from the brains of patients with Down's syndrome. Brain.

[bib6] Bishop D.V.M. (2003). The Children's Communication Checklist.

[bib7] Bishop D.V., Ross V.A., Daniels M.S., Bright P. (1996). The measurement of hand preference: a validation study comparing three groups of right-handers. British Journal of Psychology.

[bib8] Bol G., Kuiken F. (1990). Grammatical analysis of developmental language disorders: A study of the morphosyntax of children with specific language disorders, with hearing impairment and with Down's syndrome. Clinical Linguistics and Phonetics.

[bib9] Bowler D.M., Cufflin J., Kiernan C. (1985). Dichotic listening of verbal and non-verbal material by Down's syndrome children and children of normal intelligence. Cortex.

[bib10] Bruneau N., Roux S., Guerin P., Barthelemy C., Lelord G. (1997). Temporal prominence of auditory evoked potentials (N1 wave) in 4-8-year-old children. Psychophysiology.

[bib11] Bruneau N., Roux S., Adrien J.L., Barthelemy C. (1999). Auditory associative cortex dysfunction in children with autism: evidence from late auditory evoked potentials (N1 wave-T complex). Clinical Neurophysiology.

[bib12] Cacace A.T., Dowman R., Wolpaw J.R. (1988). T complex hemispheric asymmetries: effects of stimulus intensity. Hearing Research.

[bib13] Callner D.A., Dustman R.E., Madsen J.A., Schenkenberg T., Beck E.C. (1978). Life span changes in the averaged evoked responses of Down's syndrome and nonretarded persons. American Journal of Mental Deficiency.

[bib14] Carlier M., Stefanini S., Deruelle C., Volterra V., Doyen A.L., Lamard C. (2006). Laterality in persons with intellectual disability. I--do patients with trisomy 21 and Williams-Beuren syndrome differ from typically developing persons?. Behavior Genetics.

[bib15] Celesia G.G., Broughton R.J., Rasmussen T., Branch C. (1968). Auditory evoked responses from the exposed human cortex. Electroencephalography and Clinical Neurophysiology.

[bib16] Celesia G.G., Puletti F. (1969). Auditory cortical areas of man. Neurology.

[bib17] Chapman R.S. (1997). Language development in children and adolescents with Down syndrome. Mental Retardation and Developmental Disabilities Research Reviews.

[bib18] Chapman R.S., Schwartz S.E., Bird E.K. (1991). Language skills of children and adolescents with Down syndrome: I. Comprehension. Journal of Speech and Hearing Research.

[bib19] Chapman R.S., Seung H.K., Schwartz S.E., Kay-Raining Bird E. (1998). Language skills of children and adolescents with Down syndrome: II. Production deficits. Journal of Speech, Language and Hearing Research.

[bib20] Cohen J. (1992). A power primer. Psychological Bulletin.

[bib21] Connolly J.F. (1985). Stability of pathway-hemisphere differences in the auditory event-related potential (ERP) to monaural stimulation. Psychophysiology.

[bib22] Connolly J.F. (1993). The influence of stimulus intensity, contralateral masking and handedness on the temporal N1 and the T complex components of the auditory N1 wave. Electroencephalography and Clinical Neurophysiology.

[bib23] Cornish K.M., Pigram J., Shaw K. (1997). Do anomalies of handedness exist in children with fragile-X syndrome?. Laterality.

[bib24] Cracco R.Q., Amassian V.E., Maccabee P.J., Cracco J.B. (1989). Comparison of human transcallosal responses evoked by magentic coil and electrical stimulation. Electroencephalography and Clinical Neurophysiology.

[bib25] Dinani S., Carpenter S. (1990). Down's syndrome and thyroid disorder. Journal of Mental Deficiency Research.

[bib26] Dodd B. (1976). A comparison of the phonological systems of mental age matched, normal, severely subnormal and Down's syndrome children. British Journal of Disorders in Communication.

[bib27] Dodd B., Hua Z., Crosbie S., Holm A., Ozanne A. (2002). Diagnostic Evaluation of Articulation and Phonology.

[bib28] Dodd B., Thompson L. (2001). Speech disorder in children with Down's syndrome. Journal of Intellectual Disability Research.

[bib29] Dunn L.M., Dunn L.M., Whetton C., Burley J. (1997). The British Picture Vocabulary Scale.

[bib30] Eadie P., Fey M., Douglas J., Parsons C. (2002). Profiles of grammatical morphology and sentence imitation in children with specific language impairment and Down syndrome. Journal of Speech, Language, and Hearing Research.

[bib31] Elliott D., Weeks D.J. (1993). Cerebral specialization for speech perception and movement organization in adults with Down's syndrome. Cortex.

[bib32] Eulitz C., Diesch E., Pantev C., Hampson S., Elbert T. (1995). Magnetic and electric brain activity evoked by the processing of tone and vowel stimuli. Journal of Neuroscience.

[bib33] Field A. (2005). Discovering statistics using SPSS.

[bib34] Fodale V., Mafrica F., Caminiti V., Grasso G. (2006). The cholinergic system in Down's syndrome. Journal of Intellectual Disabilities.

[bib35] Fowler A.E., Cichetti D., Beeghly M. (1990). Language abilities in children with Down syndrome: evidence for a specific syntactic delay. Children with Down Syndrome: A Developmental Perspective.

[bib36] Fowler A.E., Nadel L., Rosental E. (1995). Linguistic variability in persons with Down syndrome. Down Syndrome: Living and Learning in the Community.

[bib37] Frith U., Frith C.D. (1974). Specific motor disabilities in Down's syndrome. Journal of Child Psychology and Psychiatry.

[bib38] Gomes H., Dunn M., Ritter W., Kurtzberg D., Brattson A., Kreuzer J.A. (2001). Spatiotemporal maturation of the central and lateral N1 components to tones. Brain Research Developmental Brain Research.

[bib39] Groen M.A., Yasin I., Laws G., Barry J.G., Bishop D.V.M. (2008). Weak hand preference in children with Down syndrome is associated with language deficits. Developmental Psychobiology.

[bib40] Hackley S.A., Woldorff M.G., Hillyard S.A. (1990). Cross-modal selective attention effects on retinal, myogenic, brainstem, and cerebral evoked potentials. Psychophysiology.

[bib41] Hall D.A., Johnsrude I.S., Haggard M.P., Palmer A.R., Akeroyd M.A., Summerfield Q. (2002). Spectral and temporal processing in human auditory cortex. Cerebral Cortex.

[bib42] Hartley X.Y. (1981). Lateralisation of speech stimuli in young Down's syndrome children. Cortex.

[bib43] Hill P.R., Hogben J.H., Bishop D.M. (2005). Auditory frequency discrimination in children with specific language impairment: a longitudinal study. Journal of Speech Language and Hearing Research.

[bib44] Ivarsson S.A., Ericsson U.B., Gustafsson J., Forslund M., Vegfors P., Anneren G. (1997). The impact of thyroid autoimmunity in children and adolescents with Down syndrome. Acta Paediatrica.

[bib45] Kimura D. (1967). Functional asymmetry in the brain in dichotic listening. Cortex.

[bib46] Kinsbourne M., Kinsbourne M. (1978). Biological determinants of functional bisymmetry and asymmetry. Asymmetrical Function of the Brain.

[bib47] Krapfenbauer K., Yoo B.C., Kim S.H., Cairns N., Lubec G. (2001). Differential display reveals downregulation of the phospholipid transfer protein (PLTP) at the mRNA level in brains of patients with Down syndrome. Life Science.

[bib48] Kraus N., McGee T. (1993). Clinical implications of primary and nonprimary pathway contributions to the middle latency response generating system. Ear and Hearing.

[bib49] Laws G. (2004). Contributions of phonological memory, language comprehension and hearing to the expressive language of adolescents and young adults with Down syndrome. Journal of Child Psychology and Psychiatry.

[bib50] Laws G., Bishop D.V. (2003). A comparison of language abilities in adolescents with Down syndrome and children with specific language impairment. Journal of Speech Language and Hearing Research.

[bib51] Laws G., Bishop D. (2004). Pragmatic language impairment and social deficits in Williams syndrome: a comparison with Down's syndrome and specific language impairment. International Journal of Language and Communication Disorders.

[bib52] Luck S.J. (2005). An Introduction to the Event-Related Potential Technique.

[bib53] Marcell M.M., Cohen S. (1992). Hearing abilities of Down syndrome and other mentally handicapped adolescents. Research in Developmental Disabilities.

[bib54] Mason S.M., Mellor D.H. (1984). Brain-stem, middle latency and late cortical evoked potentials in children with speech and language disorders. Electroencephalography and Clinical Neurophysiology.

[bib55] McArthur G.M., Bishop D.V.M. (2004). Which people with specific language impairment have auditory processing deficit?. Cognitive Neuropsychology.

[bib56] McCallum W.C., Curry S.H. (1980). The form and distribution of auditory evoked potentials and CNVs when stimuli and responses are lateralized. Progress in Brain Research.

[bib57] Miller J.F., Nadel L. (1988). The development of asynchrony of language development in children with Down syndrome. The Psychobiology of Down Syndrome.

[bib58] Muter V., Hulme C., Snowling M.J. (1997). The Phonological Abilities Test.

[bib59] Nehlig A., Daval J.L., Debry G. (1992). Caffeine and the central nervous system: mechanisms of action, biochemical, metabolic and psychostimulant effects. Brain Research Brain Research Reviews.

[bib60] Oerbeck B., Reinvang I., Sundet K., Heyerdahl S. (2007). Young adults with severe congenital hypothyroidism: cognitive event related potentials (ERPs) and the significance of an early start of thyroxine treatment. Scandinavian Journal of Psychology.

[bib61] Peronnet F., Michel F., Echallier J.F., Girod J. (1974). Coronal topography of human auditory evoked responses. Electroencephalography and Clinical Neurophysiology.

[bib62] Pinter J.D., Eliez S., Schmitt J.E., Capone G.T., Reiss A.L. (2001). Neuroanatomy of Down's syndrome: a high-resolution MRI study. American Journal of Psychiatry.

[bib63] Pipe M.E. (1983). Dichotic listening performance following auditory discrimination training in Down's syndrome and developmentally retarded children. Cortex.

[bib64] Ponton C., Eggermont J.J., Khosla D., Kwong B., Don M. (2002). Maturation of human central auditory system activity: separating auditory evoked potentials by dipole source modeling. Clinical Neurophysiology.

[bib65] Prasher V., Gomez G. (2007). Natural history of thyroid function in adults with Down syndrome--10-year follow-up study. Journal of Intellectual Disability Research.

[bib66] Rauschecker J.P. (1998). Parallel processing in the auditory cortex of primates. Audiology and Neurootology.

[bib67] Roid G.H., Miller L.J. (1997). Leiter International Performance Scale-Revised.

[bib68] Roizen N.J., Wolters C., Nicol T., Blondis T.A. (1993). Hearing loss in children with Down syndrome. Journal of Pediatrics.

[bib69] Rosenzweig M.R. (1951). Representation of the two ears at the auditory cortex. American Journal of Physiology.

[bib70] Schafer E.W., Peeke H.V. (1982). Down syndrome individuals fail to habituate cortical evoked potentials. American Journal of Mental Deficiency.

[bib71] Scherg M., Von Cramon D. (1986). Evoked dipole source potentials of the human auditory cortex. Electroencephalography and Clinical Neurophysiology.

[bib72] Scherg M., Von Cramon D.Y. (1985). Two bilateral sources of the late AEP as identified by a spatio-temporal dipole model. Electroencephalography and Clinical Neurophysiology.

[bib73] Schreiner C.E. (1998). Spatial distribution of responses to simple and complex sounds in the primary auditory cortex. Audiology and Neurootology.

[bib74] Seidl R., Kaehler S.T., Prast H., Singewald N., Cairns N., Gratzer M. (1999). Serotonin (5-HT) in brains of adult patients with Down syndrome. Journal of Neural Transmission Supplement.

[bib75] Sommer R.K., Starkey K.L. (1977). Dichotic verbal processing in Down's syndrome children having qualitatively different speech and language skills. American Journal of Mental Deficiency.

[bib76] Tallal P. (1976). Rapid auditory processing in normal and disordered language development. Journal of Speech and Hearing Research.

[bib77] Tallal P., Piercy M. (1973). Defects of non-verbal auditory perception in children with developmental aphasia. Nature.

[bib78] Tannock R., Kershner J.R., Oliver J. (1984). Do individuals with Down's syndrome possess right hemisphere language dominance?. Cortex.

[bib79] Tonnquist-Uhlen I. (1996). Topography of auditory evoked long-latency potentials in children with severe language impairment: the T complex. Acta Otolaryngologica.

[bib80] Tonnquist-Uhlen I., Ponton C.W., Eggermont J.J., Kwong B., Don M. (2003). Maturation of human central auditory system activity: the T-complex. Clinical Neurophysiology.

[bib81] Tunturi A.R. (1946). A study on the pathway from the medial geniculate body to the acoustic cortex in the dog. American Journal of Physiology.

[bib82] Vlkolinsky R., Cairns N., Fountoulakis M., Lubec G. (2001). Decreased brain levels of 2′, 3′-cyclic nucleotide-3′-phosphodiesterase in Down syndrome and Alzheimer's disease. Neurobiology and Aging.

[bib83] Wang P.P., Doherty S., Hesselink J.R., Bellugi U. (1992). Callosal morphology concurs with neurobehavioral and neuropathological findings in two neurodevelopmental disorders. Archives in Neurology.

[bib84] Wessinger C.M., VanMeter J., Tian B., Van Lare J., Pekar J., Rauschecker J.P. (2001). Hierarchical organization of the human auditory cortex revealed by functional magnetic resonance imaging. Journal of Cognitive Neuroscience.

[bib85] Wisniewski K.E., Schmidt-Sidor B. (1989). Postnatal delay of myelin formation in brains from Down syndrome infants and children. Clinical Neuropathology.

[bib86] Wolpaw J.R., Penry J.K. (1975). A temporal component of the auditory evoked response. Electroencephalography and Clinical Neurophysiology.

[bib87] Wolpaw J.R., Penry J.K. (1978). Effects of ethanol, caffeine, and placebo on the auditory evoked response. Electroencephalography and Clinical Neurophysiology.

[bib88] Wright B.A., Lombardino L.J., King W.M., Puranik C.S., Leonard C.M., Merzenich M.M. (1997). Deficits in auditory temporal and spectral resolution in language-impaired children. Nature.

[bib89] Wunderlich J.L., Cone-Wesson B.K., Shepherd R. (2006). Maturation of the cortical auditory evoked potential in infants and young children. Hearing Research.

